# Advances in photocatalytic research on decarboxylative trifluoromethylation of trifluoroacetic acid and derivatives

**DOI:** 10.3389/fchem.2025.1602003

**Published:** 2025-05-14

**Authors:** Fang-Fang Tan, Zhan-Chao Li

**Affiliations:** ^1^ School of Chemistry and Materials, Weinan Normal University, Weinan, China; ^2^ Sustainable Materials and Chemistry, Department of Wood Technology and Wood-based Composites, University of Göttingen, Göttingen, Germany; ^3^ School of Chemistry and Environmental Engineering, Sichuan University of Science and Technology, Zigong, China; ^4^ Key Laboratories of Fine Chemicals and Surfactants in Sichuan Provincial Universities, Zigong, China

**Keywords:** photocatalysis, trifluoromethylation, trifluoroacetic acid (TFA), decarboxylation, radical

## Abstract

Trifluoromethylation stands as a pivotal technology in modern synthetic chemistry, playing an indispensable role in drug design, functional material development, and agrochemical innovation. With the growing emphasis on green chemistry principles, the pursuit of environmentally benign trifluoromethylation strategies has emerged as a critical research frontier. Trifluoroacetic acid (TFA), characterized by its cost-effectiveness, stability, and low toxicity, has become a promising alternative to conventional trifluoromethylation reagents. This review systematically summarizes advancements in photocatalytic decarboxylative trifluoromethylation using TFA and its derivatives over the past decade, focusing on three key activation mechanisms: single-electron transfer (SET), electron donor-acceptor (EDA) complex-mediated pathways, and ligand-to-metal charge transfer (LMCT). This paradigm shift is driven by the intrinsic limitations of conventional thermal decarboxylation, particularly its reliance on harsh conditions and significant environmental burdens. In contrast, photocatalytic strategies enable efficient C–CF_3_ bond construction under mild conditions, offering a modular platform for synthesizing fluorinated functional molecules. Strategic research priorities should focus on overcoming fundamental challenges, including but not limited to optimizing photosensitizer catalytic efficiency, establishing regioselective manipulation strategies, and engineering multicomponent tandem reaction systems to achieve trifluoromethylation methodologies under mild conditions. Furthermore, the integration of mechanistic investigations with artificial intelligence-driven reaction prediction will accelerate the advancement of precision trifluoromethylation technologies. This progress is anticipated to provide sustainable synthetic solutions for next-generation fluorinated pharmaceuticals and advanced functional materials, effectively bridging the innovation gap between academic research and industrial implementation.

## 1 Introduction

Trifluoromethylation plays a significant role in contemporary chemistry (Dolbier, 1996; Ni et al., 2015). Compounds containing trifluoromethyl groups (–CF_3_) exhibit unique properties and broad application potential in pharmaceuticals, pesticides, and materials science (Muller et al., 2007). For instance, the introduction of–CF_3_ groups can markedly enhance drug bioactivity, metabolic stability, and membrane permeability, offering a robust strategy for developing highly efficient and low-toxicity therapeutics (Dolbier, 1996; Ni et al., 2015; Purser et al., 2008; Muller et al., 2007). Numerous trifluoromethylation reagents have been developed and widely used for the trifluoromethylation of organic molecules, laying a solid theoretical foundation for the development of this field. Examples include trifluorohalomethanes (CF_3_I, CF_3_Br) (Guo et al., 2018; Natte et al., 2016; Mckee and Shevlin, 1985), trifluoromethanesulfonic acid derivatives (CF_3_SO_2_Cl, (CF_3_SO_2_)_2_O, CF_3_SO_2_CH(Me)COPh) (Muralirajan et al., 2021; Ouyang et al., 2018; Liu et al., 2017), Togni reagents (Mejía and Togni, 2012; Charpentier et al., 2015), Umemoto reagents (Cheng et al., 2015; Meucci et al., 2019), TMSCF_3_ (Krishnamurti et al., 2018; Sun et al., 2019), Langlois reagents (NaSO_2_CF_3_) (Wang et al., 2016; Le et al., 2017; Jiang et al., 2021), and Zn(SO_2_CF3)_2_ (O’brien et al., 2014; Fujiwara et al., 2012). However, the low atom utilization and the production of hazardous byproducts of conventional trifluoromethylation reagents restrict their use on a large scale.

TFA, a readily available and stable trifluoromethyl source, can generate CF_3_• radicals via decarboxylation with easily separable byproducts, demonstrating significant potential (Andreev et al., 2013; Arai et al., 2014; Depecker et al., 1999; Ji et al., 2019). However, the high electronegativity of fluorine atoms elevates TFA’s oxidation potential, complicating decarboxylation under mild conditions—a critical bottleneck for its widespread adoption (Andreev et al., 2013; Arai et al., 2014). Photocatalysis, particularly visible-light-driven systems, addresses this challenge by enabling reactive intermediate generation under mild conditions, thus promoting efficient and selective synthetic routes (Prier et al., 2013; Romero and Nicewicz, 2016).

**SCHEME 1:**
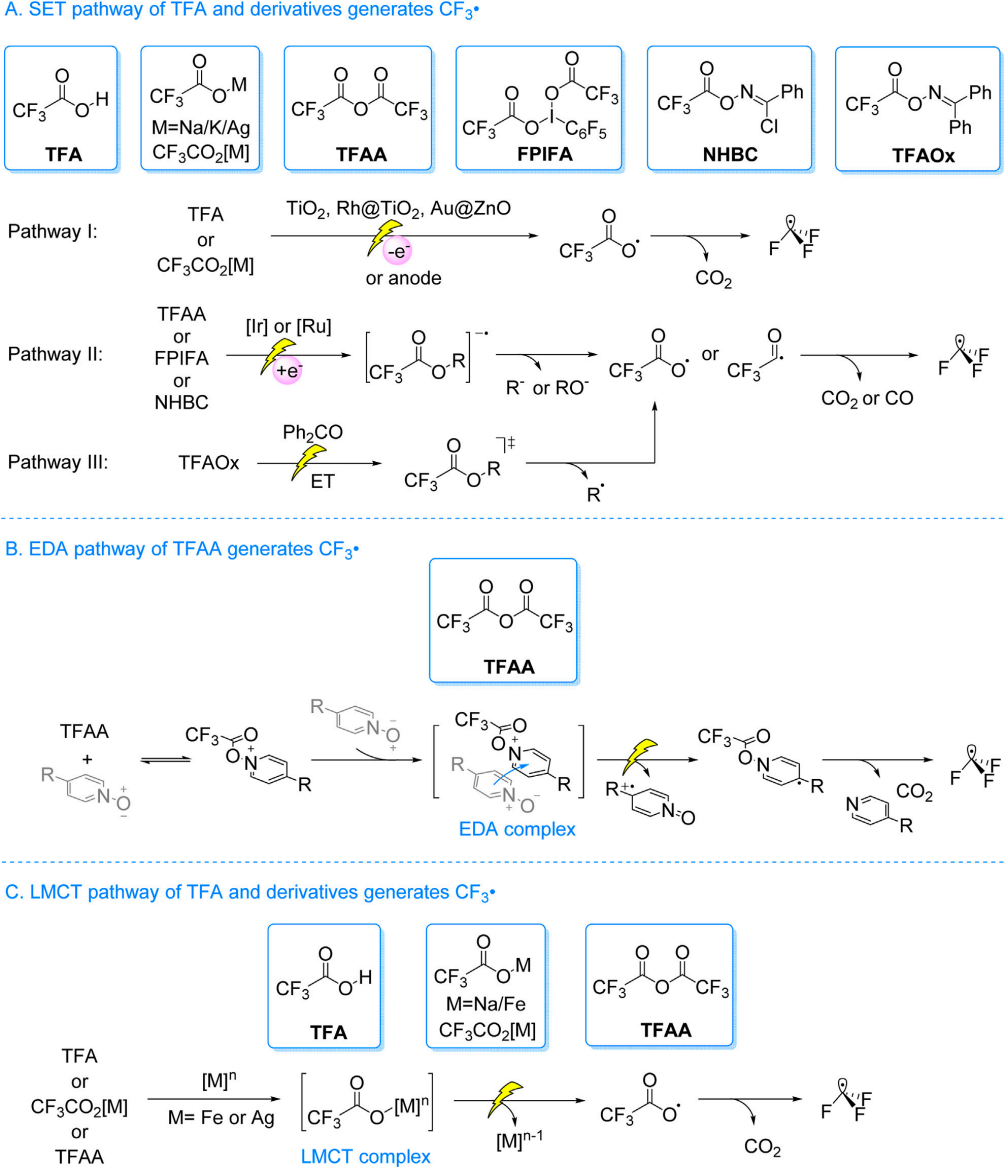
Three main routes for photocatalytic production of trifluoromethyl from trifluoroacetic acid and its derivatives.

Early studies on photocatalytic TFA decarboxylation relied on ultraviolet or high-energy tungsten lamps, or precious metal catalysts. Alternatively, researchers employed *pre*-activated or *in situ* activated derivatives to facilitate CF_3_• radicals generation under visible light. As illustrated in Scheme 1, three primary pathways exist: A) The SET process is used to generate the CF_3_• radicals. TFA or trifluoroacetate (CF_3_CO_2_[M], where M stands for Na, K or Ag) are oxidized either through holes or at the anode. This results in the formation of CF_3_CO_2_•. CO_2_ is then eliminated, resulting in CF_3_• radicals (Scheme 1A, path I). Alternatively, TFA derivatives (such as Trifluoroaceticanhydride (TFAA), pentafluoroiodobenzene bis (trifluoroacetate) (FPIFA), trifluoroacetoxybenzimidazole chloride ester (NHBC)) receive an electron from the excited photocatalyst. This results in the formation of their anionic radical intermediates, from which the anionic group (R^−^/RO^−^) departs, yielding CF_3_CO_2_• or CF_3_CO•. The subsequent loss of CO_2_ or CO then generates the CF_3_• radicals (Scheme 1A, path II). Additionally, trifluoroacetyl oxime (TFAOx) can produce CF_3_• radicals via an energy transfer (ET) process (Scheme 1A, path III); B) The EDA procedure is used to generate the CF_3_• radicals. Pyridine N-oxide reacts with TFAA to form an intermediate trifluoroacetoxypyridinium ion. This intermediate acts as an electron acceptor and forms an EDA complex with pyridine N-oxide, which serves as the electron donor. Upon exposure to visible light, electrons are transferred from the donor to the acceptor, leading to the formation of a dearomatized tertiary carbon radical intermediate. This intermediate induces homolytic cleavage of the N–O bond, yielding the CF_3_CO_2_•. Subsequent elimination of CO_2_ results in the formation of the CF_3_• radicals (Scheme 1B); C) The LMCT process is used to generate the CF_3_• radicals. The CF_3_CO_2_^−^, obtained from TFA, CF_3_CO_2_[M], or TFAA, coordinates with the metal center of a complex. Upon light excitation, the newly formed complex undergoes the LMCT process, producing the CF_3_CO_2_•. Subsequently, the elimination of CO_2_ results in the formation of the CF_3_• radicals (Scheme 1C).

The strategic incorporation of CF_3_ groups into aromatic systems has become a hallmark of modern medicinal chemistry, as evidenced by the prevalence of this motif in 7.5% of FDA-approved small-molecule drugs (2018 data), with 80% exhibiting direct aromatic-CF_3_ connectivity—a structural signature shared by therapeutic agents such as Enzalutamide (prostate cancer), Fluoxetine (depression), Lomitapide (hypercholesterolemia), and Regorafenib (leukemia). To address the growing demand for sustainable construction of these pharmacologically privileged scaffolds, this review interrogates cutting-edge advancements in visible-light-mediated trifluoromethylation of (hetero)arenes and alkenes using TFA as a bench-stable CF_3_• precursor. By deconvoluting three dominant photocatalytic mechanisms—SET, EDA complex activation, and LMCT—we establish comparative mechanistic frameworks that unify radical initiation dynamics across disparate systems. These analyses reveal design principles for optimizing radical generation efficiency and intermediate stabilization, providing actionable strategies to advance trifluoromethylation technologies toward atom-economic and operationally simple synthesis.

## 2 Photocatalytic decarboxylating trifluoromethylation of TFA and its derivatives

### 2.1 Catalyst system for SET

**SCHEME 2:**
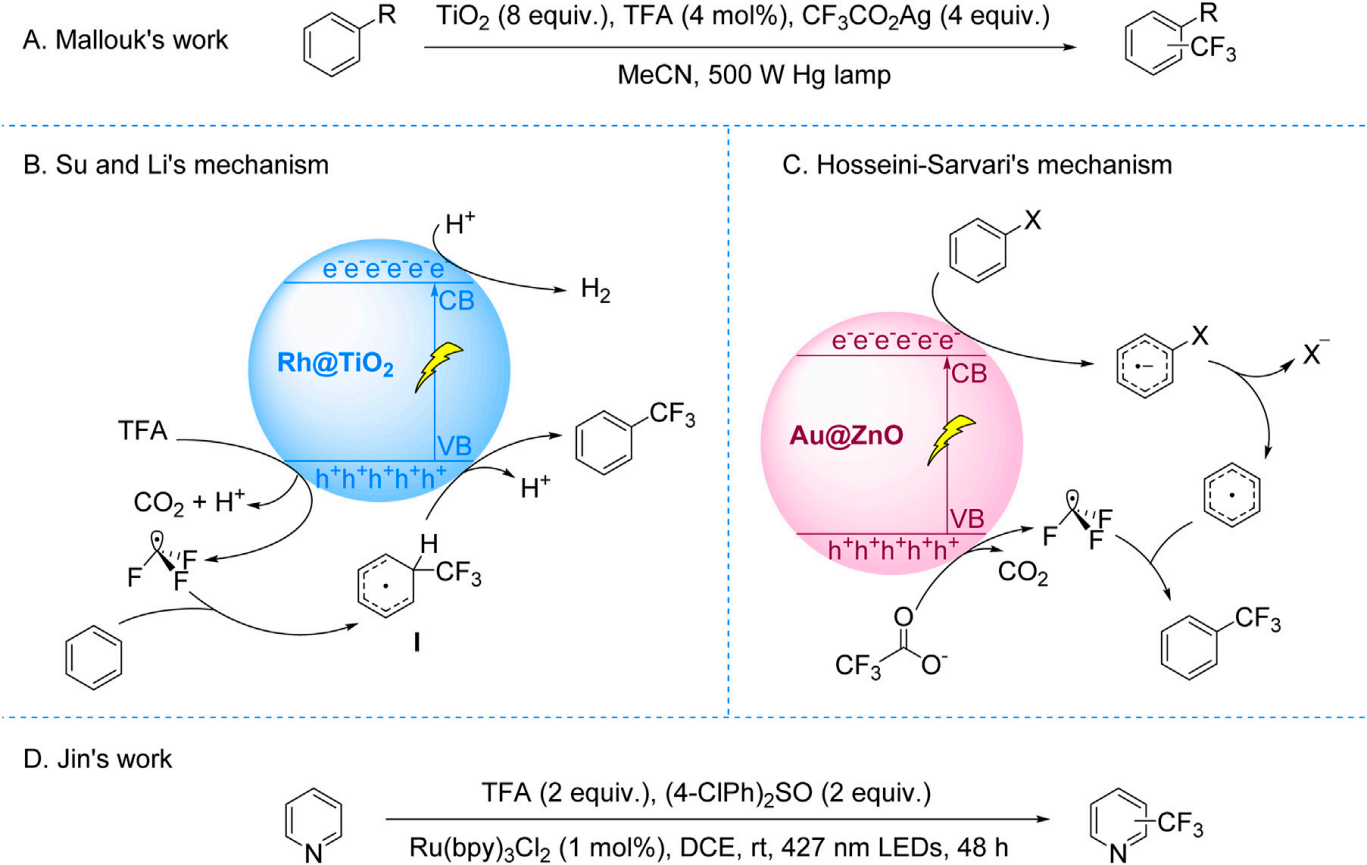
Photocatalytic decarboxylative trifluoromethylation of TFA and trifluoroacetates. **(A)** TiO_2_-catalyzed C–H trifluoromethylation of (hetero)arenes via CF_3_CO_2_Ag decarboxylation; **(B)** Rh@TiO_2_-catalyzed C–H trifluoromethylation of (hetero)arenes via TFA decarboxylation; **(C)** Au@ZnO-catalyzed trifluoromethylation of (hetero)arenes via CF_3_CO_2_Na decarboxylation; **(D)** Ru(bpy)_3_Cl_2_-catalyzed trifluoromethylation of (hetero)arenes via TFA decarboxylation.

The most frequent SET process in photoredox reactions usually entails the photocatalyst’s gain or loss of an electron. The production of active radical intermediates is facilitated by this mechanism (Prier et al., 2013; Romero and Nicewicz, 2016). Studies reveal that radicals like CF_3_CO_2_• or CF_3_CO• undergo a quick decarbonylation process. The CF_3_• radicals is produced as a result of the removal of CO_2_ or CO during this process (Sawada et al., 1990; Zhong et al., 2015). The mechanistic understanding of CF_3_• radical generation via decarboxylation or decarbonylation laid the foundation for developing photocatalytic trifluoromethylation strategies. A key milestone was achieved in 1993 when Mallouk’s group pioneered the photocatalytic trifluoromethylation of aromatic C-H bonds using CF_3_CO_2_Ag as the CF_3_ source and TiO_2_ as the catalyst under 500 W Hg lamp irradiation (Scheme 2A; Lai and Mallouk, 1993). This work marked the first demonstration of light-induced trifluoroacetate decarboxylative trifluoromethylation. However, the system suffered from metallic Ag deposition-induced catalyst deactivation, requiring excess TiO_2_ to sustain reactivity, and exhibited limited regioselectivity for substituted arenes, resulting in mixed isomer products. Mechanistic investigations, including hexafluoroethane detection and radical trapping experiments, conclusively identified CF_3_• radicals as the pivotal intermediates.

Subsequent advances addressing these challenges expanded both mechanistic understanding and substrate applicability. In 2017, Su and Li developed a Rh@TiO_2_-mediated photocatalytic system for C–H trifluoromethylation of (hetero)arenes using TFA as a CF_3_ source under 365 nm UV light. Their mechanistic studies revealed that hole-induced TFA oxidation generated CF_3_• radicals, which coupled with arenes to form intermediates subsequently oxidized to final products, concurrent with H_2_ evolution at the conduction band (Scheme 2B; Lin et al., 2017). Complementing this approach, Hosseini-Sarvari’s group employed CF_3_CO_2_Na and blue LED irradiation to achieve Au@ZnO-catalyzed trifluoromethylation of diverse C–X (X = I, Br, B) and C–H bonds (Bazyar and Hosseini-Sarvari, 2019). They proposed a dual radical pathway involving conduction band reduction-triggered aryl radical formation and valence band oxidation of CF_3_CO_2_^−^ to CF_3_•, followed by selective radical coupling (Scheme 2C).

The field’s progression toward visible-light compatibility culminated in Jin’s 2020 work employing Ru(bpy)_3_Cl_2_ and stoichiometric (4-ClPh)_2_SO to drive TFA decarboxylative trifluoromethylation under visible light (Scheme 2D). Mechanistic evidence suggested (4-ClPh)_2_SO facilitates light-induced CF_3_• generation via TFA decarboxylation, with subsequent dearomative addition to (hetero)arenes forming trifluoromethylated products. Nevertheless, the precise redox synergy between TFA and (4-ClPh)_2_SO remained unresolved. While these innovations validated TFA-based CF_3_• generation, persistent limitations—catalyst deactivation, erratic regioselectivity, and UV dependency—underscored the necessity for visible-light systems with enhanced control. This imperative catalyzed the emergence of photoelectrocatalytic paradigms that integrate optical and electrical fields to manipulate radical dynamics, offering new precision in trifluoromethylation strategies.

**SCHEME 3:**
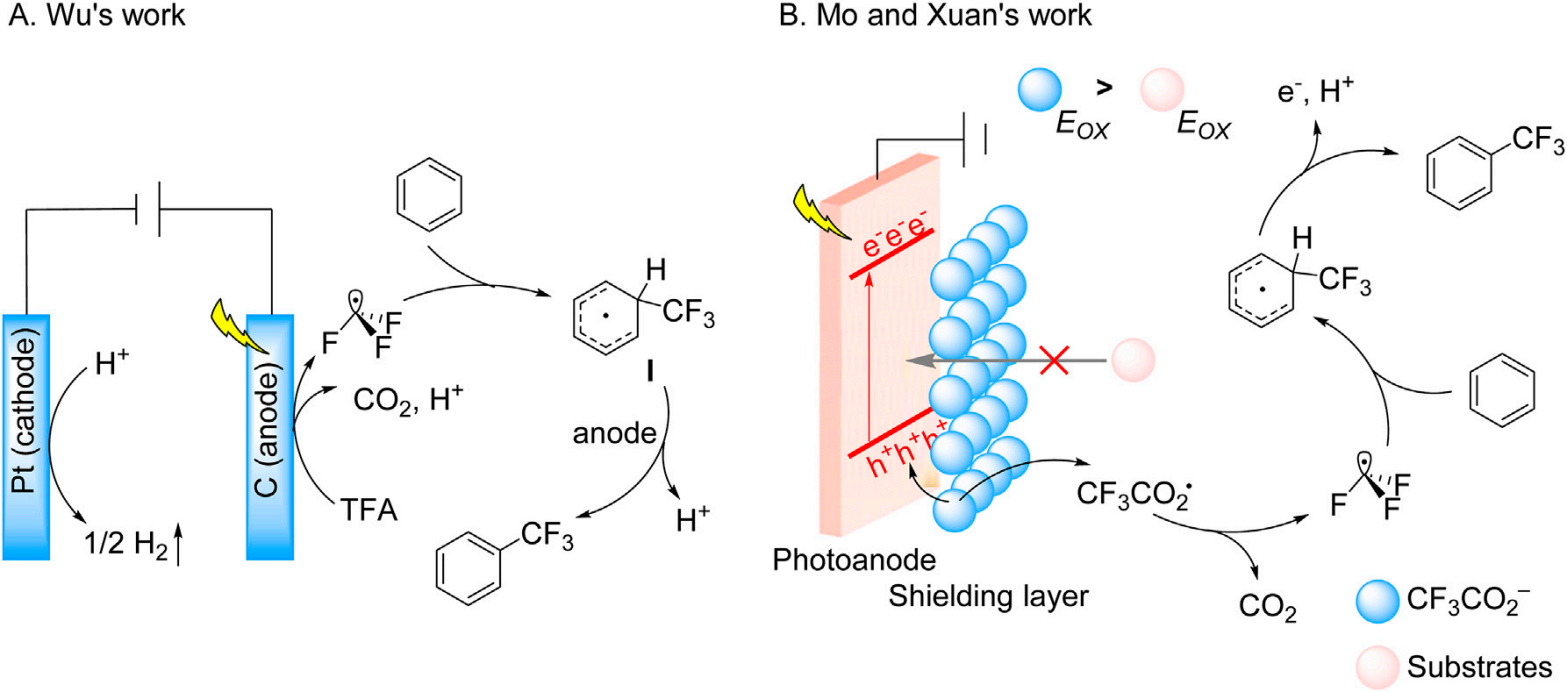
Photoelectrocatalytic decarboxylative trifluoromethylation of TFA and CF_3_CO_2_K. **(A)** Graphite anode-mediated oxidative TFA decarboxylation for (hetero)arene trifluoromethylation; **(B)** Mo-doped WO_3_ anode-mediated oxidative CF_3_CO_2_K decarboxylation for (hetero)arene trifluoromethylation.

For instance, the Wu group reported a photoelectrocatalytic platform for direct (hetero)arene C-H trifluoromethylation using TFA, employing a graphite-Pt dual-electrode system under 5 mA current and 390 nm LED irradiation (Qi et al., 2023). This system addressed the scalability challenge by enabling continuous operation without catalyst degradation. Although effective for electron-deficient aromatics, regioselectivity limitations arose from radical-mediated pathways. Mechanistic studies revealed that photoinduced electron-hole pairs at the graphite anode selectively oxidize CF_3_CO_2_^−^ through electrostatic interactions, preserving neutral arenes. The generated CF_3_• radicals undergo dearomative addition to form cyclohexadienyl intermediates, which are subsequently oxidized and deprotonated to regenerate aromaticity, with concurrent H_2_ evolution at the Pt cathode ensuring charge balance (Scheme 3A). This dual-functional design not only resolved catalyst deactivation but also facilitated late-stage fluorofunctionalization of pharmaceuticals and natural products, demonstrating versatile synthetic applicability.

Building on this, Mo and Xuan introduced an ion-shielded photoelectrocatalytic strategy to address thermodynamic bottlenecks in C–H trifluoromethylation (Chen et al., 2024). Utilizing a Mo-doped WO_3_ photoanode under 390 nm irradiation with a 2 V bias, CF_3_CO_2_^−^ anions formed an electrostatic shielding layer on the anode surface. This layer selectively blocked substrate access to photogenerated holes while promoting preferential oxidation of CF_3_CO_2_^−^ to CF_3_• radicals. These radicals engaged (hetero)arenes via sequential oxidation-dearomatization-deprotonation cascades (Scheme 3B). This study elucidates the mechanistic paradigm for enabling selective electron transfer under extreme anodic polarization while establishing an operationally scalable platform for trifluoromethylation processes.

**SCHEME 4:**
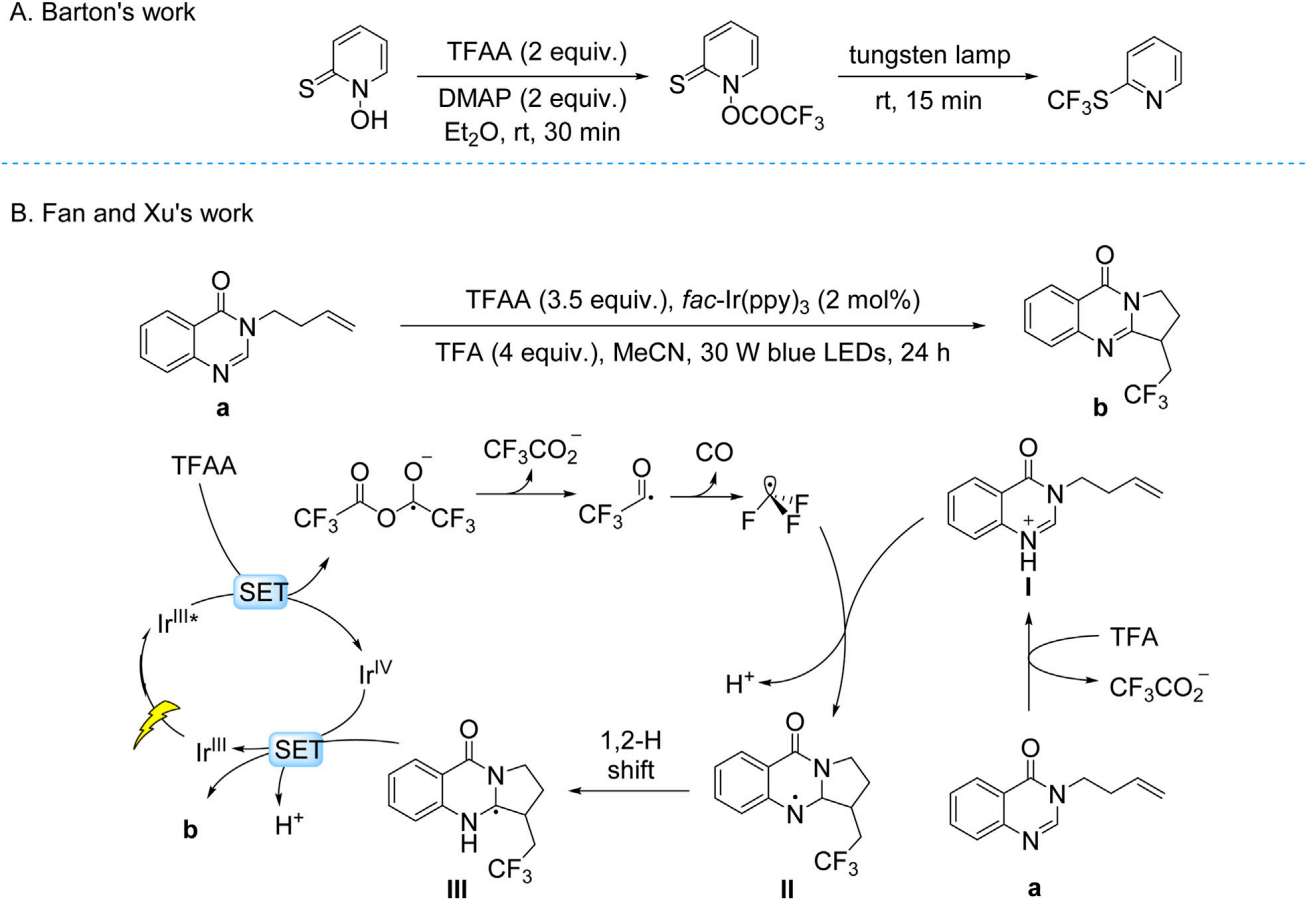
Photocatalytic decarboxylative trifluoromethylation of TFAA. **(A)** Trifluoromethylation of 1-hydroxypyridine-2-thione in the early phase using TFAA as a trifluoromethyl source; **(B)** fac-Ir(ppy)_3_-catalyzed trifluoromethylation using TFAA as the trifluoromethyl source.

While photoelectrocatalysis has opened new mechanistic avenues for trifluoromethylation, conventional photocatalytic approaches have simultaneously advanced through systematic engineering of reaction systems. The foundations for these developments were established by early photoredox studies, particularly the seminal 1986 report by Barton and coworkers, who achieved the trifluoromethylation of 1-hydroxypyridine-2-thione using TFAA as the CF_3_ source (Scheme 4A; Barton et al., 1986). This transformation, which proceeds via light-induced decarboxylation and rearrangement, represented a pioneering example of radical-mediated C–CF_3_ bond formation. Building upon these fundamental insights, contemporary researchers have developed more sophisticated photocatalytic systems. The Pan group recently demonstrated an efficient, additive-free photoredox protocol utilizing TFAA for the trifluoromethylation of (hetero)aromatics and polarized alkenes (Song et al., 2023). This method exhibits remarkable substrate scope, accommodating natural product derivatives and pharmaceutical scaffolds, while mechanistic studies confirmed the generation of CF_3_• radicals through visible-light-driven activation of TFAA.

Expanding the synthetic utility of this approach, Fan and Xu group developed a visible-light-mediated tandem trifluoromethylation/cyclization of quinazolinone-tethered alkenes (Zou et al., 2024). Employing fac-Ir(ppy)_3_ irradiation at 457 nm, this transformation proceeds without strong oxidants to construct tri- and tetracyclic quinazolinones with excellent functional group tolerance. Detailed mechanistic investigations revealed a sequence involving: (i) photoinduced single-electron transfer to TFAA, generating a radical anion that fragments to form CF_3_CO_2_•; (ii) decarbonylation to CF_3_• radicals; and (iii) radical addition followed by cyclization through deprotonation, hydrogen migration, and aromatization steps (Scheme 4B).

**SCHEME 5:**
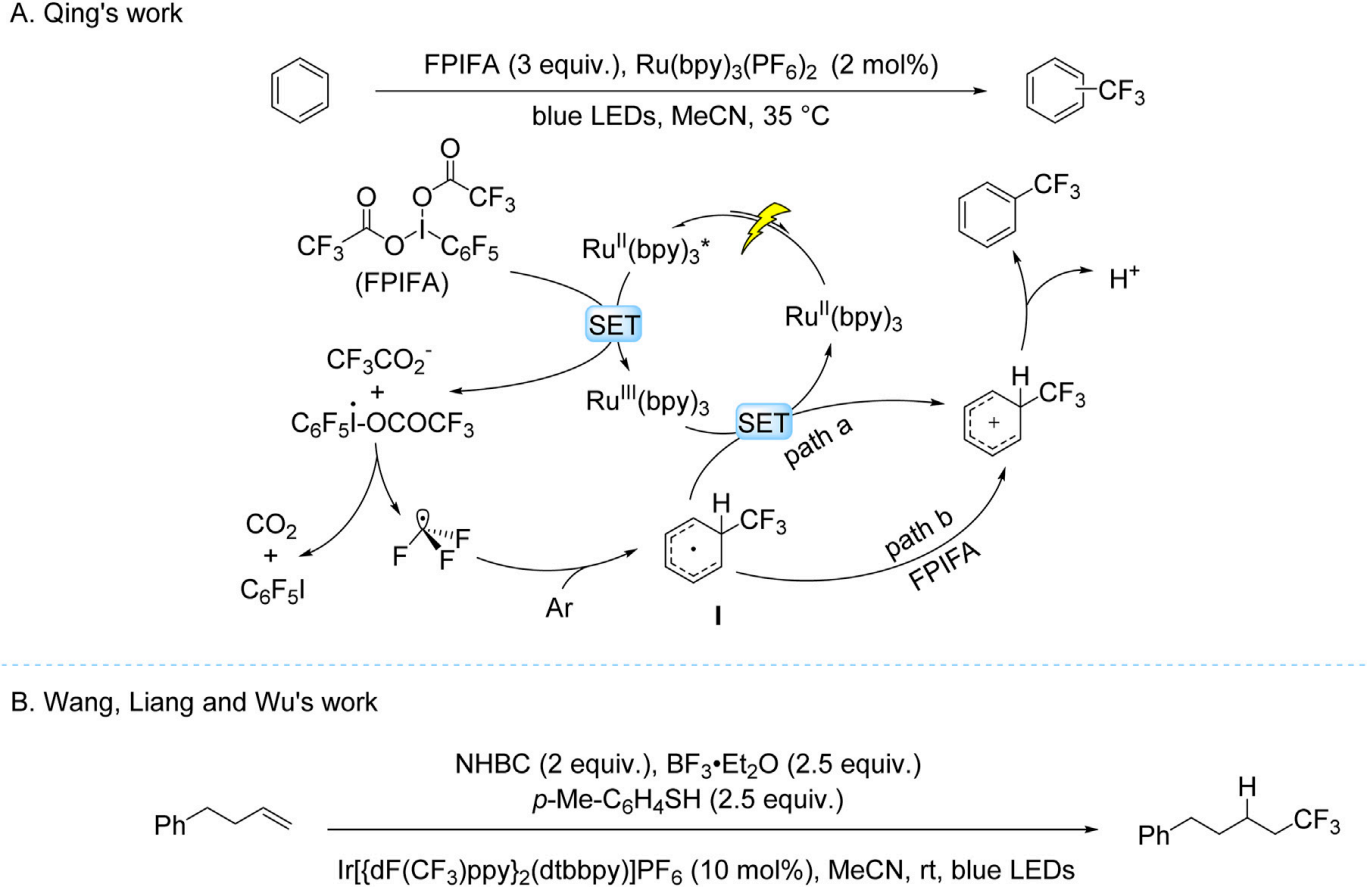
Photocatalytic single-electron oxidation of TFA derivatives. **(A)** Ru(bpy)_3_(PF_6_)_2_-catalyzed trifluoromethylation using FPIFA as the trifluoromethyl source; **(B)** based-Ir-catalyzed trifluoromethylation using NHBC as the trifluoromethyl source.

These seminal works on TFAA activation exemplify the remarkable progress in photocatalytic trifluoromethylation, yet the field continues to evolve through innovative system design and mechanistic understanding. A prime example of such advancement emerged from the Qing group’s 2018 work, which demonstrated visible-light-driven C–H trifluoromethylation of electron-deficient arenes using Ru(bpy)_3_(PF_6_)_2_ and FPIFA (Yang et al., 2018). The protocol operated under mild conditions, delivering moderate-to-good yields albeit with limited regioselectivity. Mechanistic studies revealed that oxidative quenching of the photoexcited Ru(II)* species by FPIFA generated Ru(III) and a hypervalent iodine intermediate, which subsequently decomposed to release CF_3_• radicals. These radicals underwent dearomatization with the arene substrate, forming a cyclohexadienyl radical intermediate. This intermediate followed dual oxidation pathways (mediated by Ru(III) or FPIFA) and subsequent deprotonation to yield the trifluoromethylated product (Scheme 5A). Further expanding the reaction scope, Wang, Liang and Wu subsequently developed a hydrotrifluoromethylation protocol for unactivated alkenes using NHBC as a radical precursor (Scheme 5B; Zhang et al., 2019). The proposed mechanism involves oxidative quenching of the photoexcited Ir(III) photocatalyst by NHBC, generating a CF_3_CO_2_• that undergoes decarboxylation to produce CF_3_• radicals. These radicals add to alkenes, followed by HAT-mediated hydrogen abstraction to afford the final hydrotrifluoromethylated product.

**SCHEME 6:**
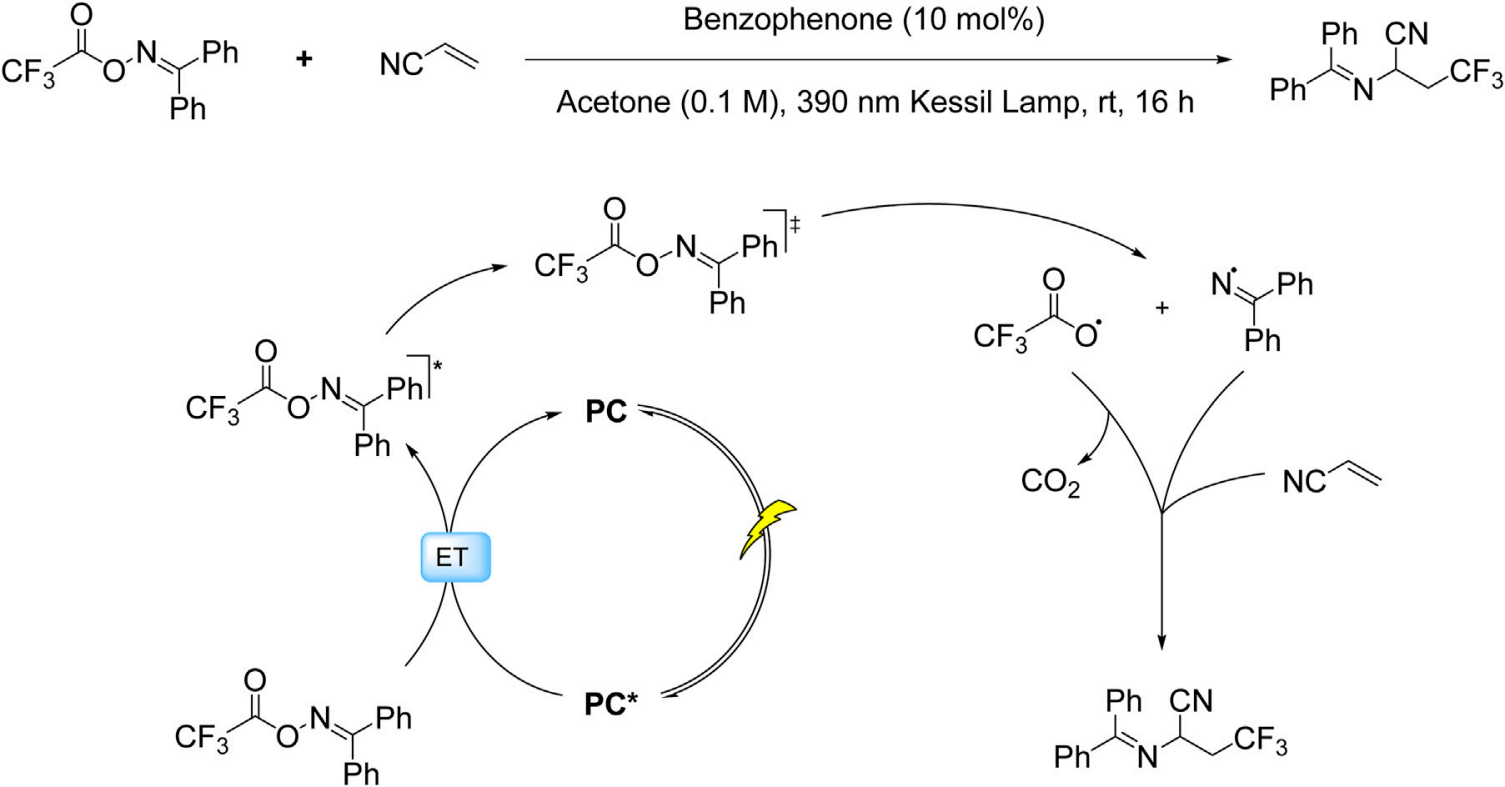
Photocatalytic ET-mediated decarboxylative trifluoromethylation of alkenes via TFAOx activation.

While SET processes have dominated photoredox trifluoromethylation strategies, recent advances have unveiled the complementary potential of ET mechanisms in radical generation. This paradigm shift is exemplified by the Molander group’s innovative work employing benzophenone-mediated triplet-triplet energy transfer to activate trifluoroacetyl oxime esters (TFAOx) (Majhi et al., 2022). In contrast to conventional SET pathways that rely on redox potentials, this ET approach operates through direct photoexcitation of the oxime ester to its triplet state, triggering N–O bond homolysis and subsequent decarboxylation to generate both CF_3_• and persistent iminyl radicals. The CF_3_• radicals undergo kinetically favorable, irreversible and regioselective addition to terminal alkene positions, while the iminyl radicals propagate a chain cycle through interaction with additional oxime ester molecules, ultimately enabling synergistic C–C/C–N bond formation (Scheme 6).

### 2.2 Catalyst system for EDA

EDA complexes, formed through non-covalent interactions between electron-rich donors (D) and electron-deficient acceptors (A), enable directional electron transfer under photoexcitation to generate solvent-caged radical ion pairs, which drive diverse reaction pathways such as radical recombination and polarity inversion (Lee and Kochi, 1992; Lima et al., 2016). This mechanism underpins versatile applications in organic synthesis, including catalytic alkylation of carbonyl compounds and stereoselective construction of complex architectures. The EDA paradigm exemplifies a sustainable strategy for achieving spatiotemporal control over reactivity, offering transformative potential for green synthesis of high-value molecules.

**SCHEME 7:**
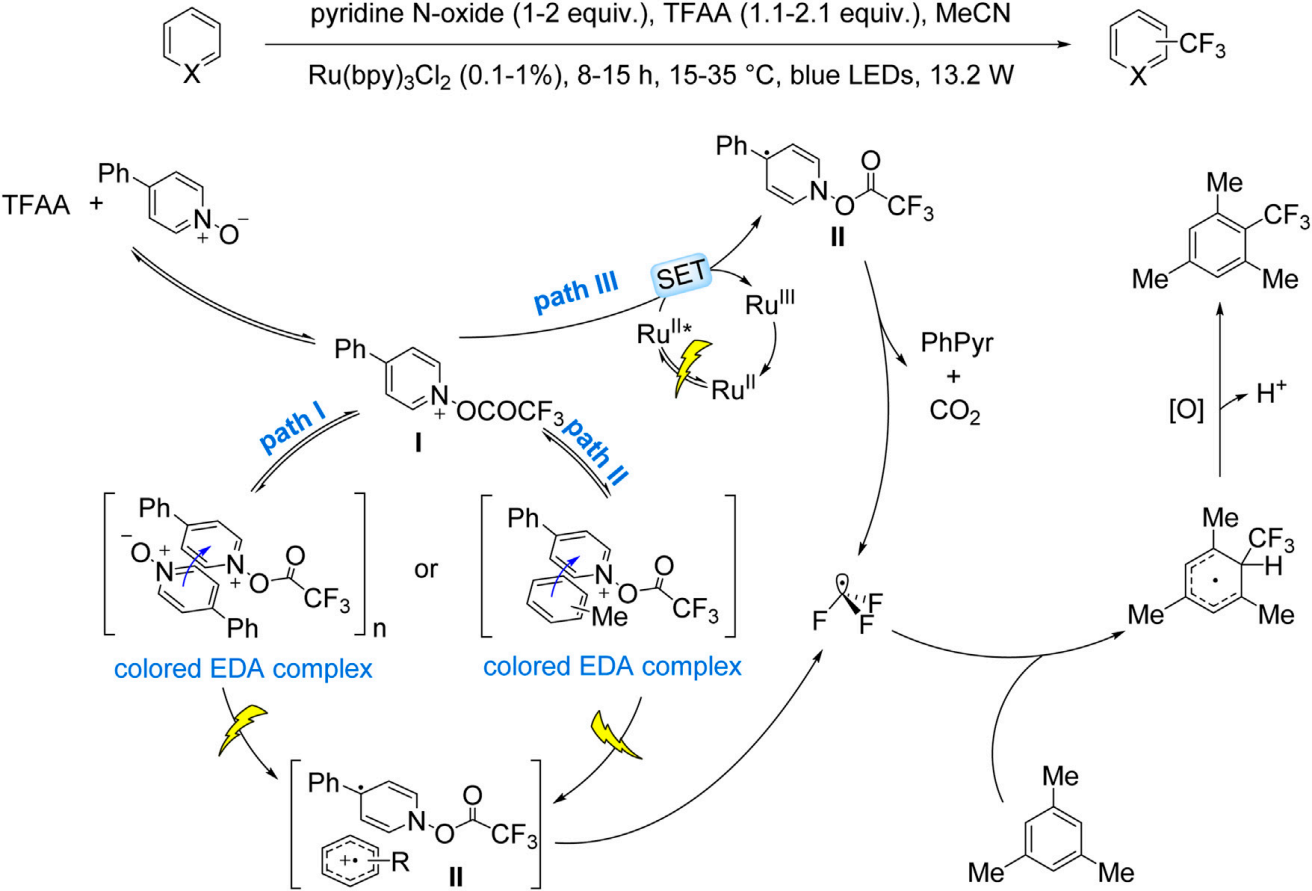
Stephenson’s pioneering work on EDA-mediated trifluoromethylation.

The Stephenson group pioneered this approach in 2015, reporting the trifluoromethylation of (hetero)arene C–H bonds using TFAA as the CF_3_ source, Ru(bpy)_3_Cl_2_·6H_2_O as the photocatalyst, and pyridine *N*-oxide as the oxidant, with pyridine generated *in situ* as the base (Beatty et al., 2015). This catalytic system exhibited operational simplicity and scalability to gram-scale synthesis, albeit with moderate regioselectivity and yields. In 2016, the same group expanded on this work, elucidating the mechanism and broadening the substrate scope to include complex (hetero)aromatics, highlighting its potential for pharmaceutical synthesis. Mechanistic studies revealed the formation of a 4-phenyl-1-(trifluoroacetoxy)pyridinium intermediate (I) from TFAA and 4-phenylpyridine *N*-oxide. Intermediate **I** generates CF_3_• radicals through three distinct pathways: (i) EDA complex formation with 4-phenylpyridine *N-*oxide or aromatic substrates, followed by photoinduced electron transfer to yield radical intermediate **II** and subsequent release of 4-phenylpyridine and CO_2_ (Scheme 7, Paths I and II); (ii) Intermediate **I** oxidizes Ru(II)* via a SET process, forming intermediate **II** and Ru(III) while releasing 4-phenylpyridine and CO_2_ simultaneously (Scheme 7, Path III). The resulting CF_3_• radicals undergo dearomatization via addition to arenes, forming cyclohexadienyl intermediates that re-aromatize upon oxidation to afford the final products (Beatty et al., 2016).

**SCHEME 8:**
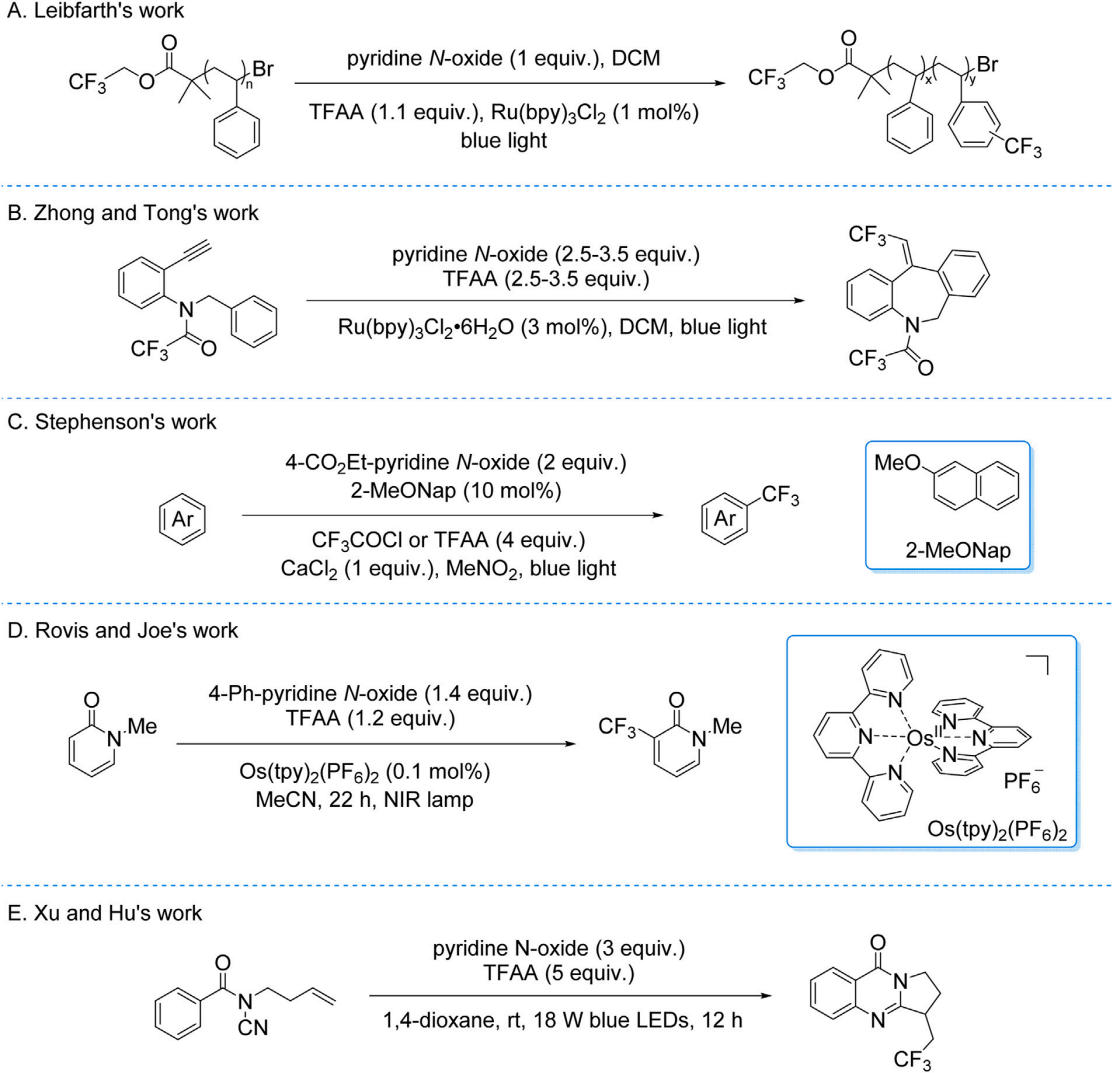
Photocatalytic generation of CF_3_• radicals from TFA and derivatives via the EDA process. **(A)** Leibfarth’s expansion to industrial applications; **(B)** Advancements in tandem trifluoromethylation and cyclization; **(C)** Stephenson’s redesign of the EDA platform; **(D)** Application of Os(tpy)_2_(PF_6_)_2_ as a near-infrared photocatalyst in EDA processes; **(E)** Tandem trifluoromethylation-cyclization reaction.

Building on these foundations, the Leibfarth group adapted the system in 2019 for the trifluoromethylation of industrial plastic waste and commercial plastics (Scheme 8A; Lewis et al., 2019). This work extended the synthetic utility of photocatalytic C–H trifluoromethylation to polymer functionalization, offering a potential strategy for upcycling plastic materials. Concurrently, Zhong and Tong developed a photocatalytic system for the tandem trifluoromethylation and cyclization of secondary benzylamines using TFAA/pyridine *N*-oxide/Ru(bpy)_3_Cl_2_ (Scheme 8B). Mechanistic studies proposed that photoinduced generation of CF_3_• radicals—via EDA complex-mediated or SET pathways—initiated the transformation. This method demonstrated precise control over regioselectivity in complex heterocycle synthesis (Qi et al., 2019).

In 2020, the Stephenson group redesigned their EDA platform to mitigate back-electron transfer in homogeneous photocatalysis (Scheme 8C; Mcclain et al., 2020). By replacing Ru(bpy)_3_Cl_2_ with a dual-component EDA system (2-methoxynaphthalene donor + *in situ*-generated acylated 4-ethoxycarbonylpyridine *N-*oxide acceptor from CF_3_COCl/TFAA), they achieved photosensitizer-free CF_3_• radicals generation. EDA complex photoexcitation triggered charge transfer, yielding a 2-methoxynaphthalene radical cation and releasing CF_3_• radicals via acceptor decarboxylation. The radicals engaged in Minisci-type C–H trifluoromethylation of (hetero)arenes, forming a dearomatized intermediate rearomatized by the radical cation to close the catalytic cycle. This strategy achieved moderate yields while eliminating traditional photosensitizers, though catalytic electron donor quantities remained essential. The work demonstrates charge-transfer system engineering to suppress parasitic electron loss, advancing practical late-stage aromatic C–H trifluoromethylation. Furthermore, the Rovis and Joe’s group developed a catalytic system for the trifluoromethylation of 1-methylpyridine-2(1H)-one to synthesize 1-methyl-3-(trifluoromethyl)pyridine-2(1H)-one, employing TFAA/pyridine *N*-oxide and Os(tpy)_2_(PF_6_)_2_ as the photocatalyst (Scheme 8D). The Os(tpy)_2_(PF_6_)_2_ complex uniquely harnesses near-infrared (NIR) and deep-red (DR) light with minimal energy loss, addressing the ∼25% energy dissipation observed in conventional Ru/Ir-based systems during excited-state quenching (Ravetz et al., 2020).

In 2022, Xu, Hu, et al. developed a photoinduced radical tandem trifluoromethylation/cyclization strategy using TFAA activated by pyridine *N*-oxide (Scheme 8E; Hu et al., 2022). This methodology leverages an EDA process to generate CF_3_• radicals, which are captured by *N*-cyanamide olefins, yielding diverse trifluoromethylated quinazolinones with high efficiency and broad functional group tolerance. While photocatalytic trifluoromethylation strategies exploiting EDA complex mechanisms enable precise C–H activation and heterocycle construction under mild conditions—advancing sustainable fluorofunctionalization for pharmaceutical late-stage modification and complex molecule synthesis—their applicability remains constrained by inherent substrate scope limitations of EDA-mediated processes.

### 2.3 Catalyst system for LMCT

The LMCT process involves electron transfer from ligand orbitals to empty metal center orbitals, facilitating electron migration from the ligand to the metal center (Juliá, 2022). This process typically occurs in complexes with electrophilic, high-valent metal centers (e.g., Fe(III), Ag(II)) due to the reduced energy barrier for electron transfer. Electron-rich *σ-* or *σ/π*-hybridized ligands are more prone to LMCT transitions as they act as intrinsic electron donors (Juliá, 2022). This unique electron transfer mode has opened new pathways for chemical transformations under mild conditions and has been utilized in the trifluoromethylation of organic molecules via TFA decarboxylation (Liu et al., 2024). Mechanistically, a metal catalyst forms a complex with CF_3_CO_2_^−^, which undergoes the LMCT process under visible light to generate CF_3_CO_2_•. These radicals drive *β*-bond cleavage and subsequent transformations, underscoring the potential of LMCT in catalyzing decarboxylative trifluoromethylation of TFA and its salts.

**SCHEME 9:**
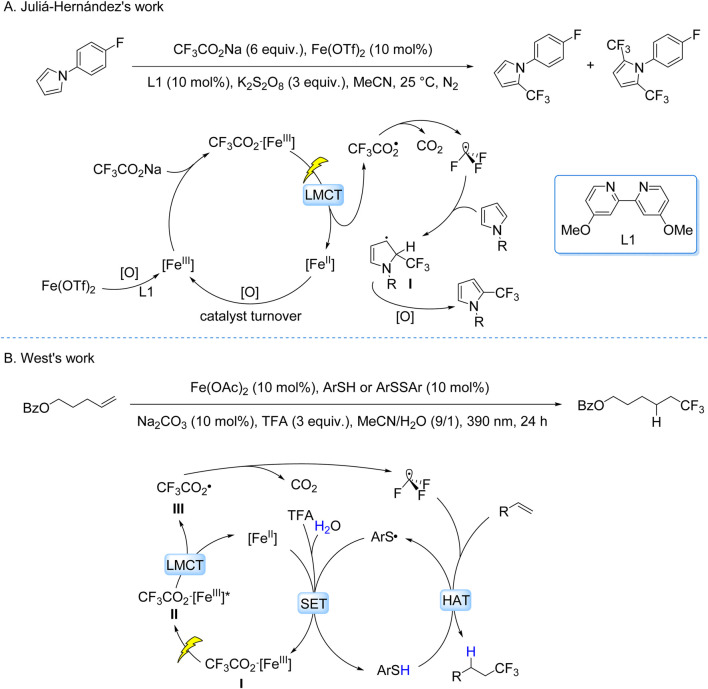
Photocatalytic generation of •CF_3_ from TFA and derivatives via the LMCT process. **(A)** Fe(AcO)_2_-catalyzed generation of •CF_3_ from CF_3_CO_2_Na via LMCT process; **(B)** Ag(bpy)_2_(OTf)_2_ -catalyzed generation of •CF_3_ from CF_3_CO_2_Na via LMCT process.

The Juliá-Hernández group developed a visible-light-driven trifluoromethylation of (hetero)arenes using CF_3_CO_2_Na as the CF_3_ source and an Fe(OTf)_2_/4,4′-dimethoxy-2,2′-bipyridine/K_2_S_2_O_8_ system under 405 nm irradiation (Scheme 9A; Fernandez-Garcia et al., 2024). Mechanistic studies revealed a LMCT mechanism: Fe-ligand complexes underwent photoinduced O–Fe bond homolysis, generating CF_3_CO_2_• radicals that decarboxylated to CF_3_• radicals. These radicals participated in Minisci-type addition to electron-rich (hetero)aromatics, forming intermediates oxidized to final products. The protocol demonstrated broad substrate tolerance, including pyrroles, indoles, and bioactive molecules, with moderate to good yields. However, regioselectivity remained limited for polyfunctional substrates, reflecting inherent challenges in controlling radical pathways in complex systems.

The West group developed a redox-neutral hydrofluoroalkylation of alkenes under mild conditions, employing TFA as the CF_3_ source, Fe(OAc)_2_ as the catalyst, aryl thiols (ArSH) or disulfides (ArSSAr) as HAT mediators, Na_2_CO_3_ as the base, and 390 nm light irradiation (Bian et al., 2023). This dual catalytic system synergizes LMCT and HAT pathways through four key stages: (i) photolytic cleavage of the O–Fe bond in the Fe(II)–TFA coordination complex generates CF_3_CO_2_• concurrent with reduction of Fe(III) to Fe(II); (ii) decarboxylation of CF_3_CO_2_• produces CF_3_• radicals; (iii) radical addition to alkenes forms transient carbon-centered radical intermediates; (iv) HAT from ArSH to the intermediate delivers hydrofluoroalkylated products (Scheme 9B). This mechanistically integrated platform enables late-stage installation of C–CF_3_ bonds in structurally complex bioactive molecules, demonstrating compatibility with over twenty functional groups including electrophilic and protic moieties.

**SCHEME 10:**
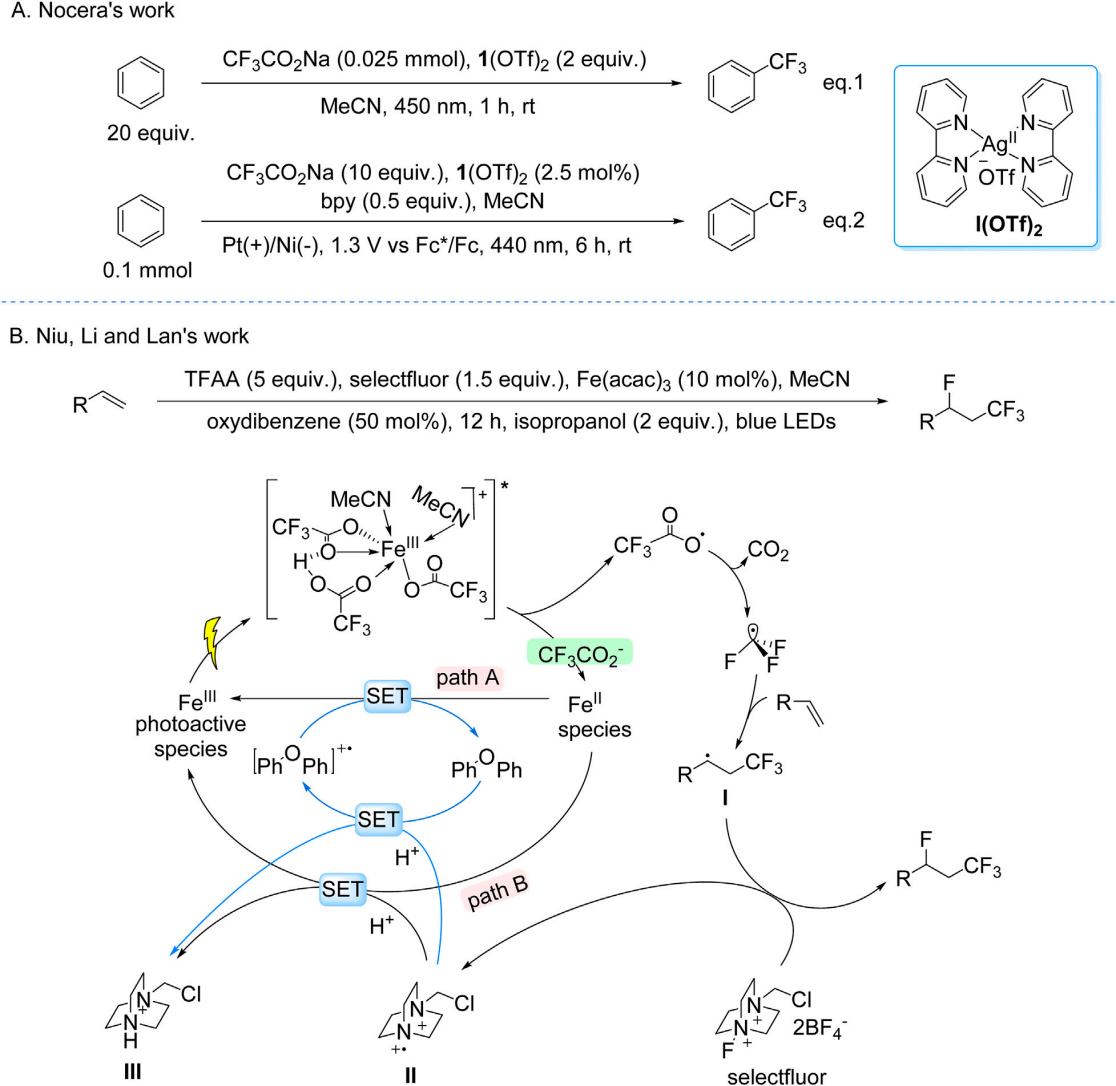
Photocatalytic generation of •CF_3_ from TFA and derivatives via the LMCT process. **(A)** Ag(OTf)_2_-catalyzed generation of •CF_3_ from TFA via LMCT process; **(B)** Fe(acac)_3_-catalyzed generation of •CF_3_ from TFAA via LMCT process.

The Nocera group developed a dual-mode catalytic strategy for arene trifluoromethylation using Ag(bpy)_2_(OTf)_2_ and CF_3_CO_2_Na under visible light irradiation. The reaction proceeds via a LMCT mechanism: photoexcitation of the electrophilic Ag(II) center in the Ag(II)–CF_3_CO_2_^−^ coordination complex induces O–Ag bond homolysis, generating CF_3_• radicals. Two distinct operational modes were engineered—(i) a stoichiometric photocatalytic system (2.0 equiv Ag) to offset incomplete Ag(I)-to-Ag(II) reoxidation, and (ii) a catalytic photoelectrochemical system (2.5 mol% Ag) leveraging anodic oxidation to regenerate Ag(II) from Ag(I), enabling sustained turnover. The CF_3_• radicals engage in dearomatizing addition to arenes, followed by Ag(II)-mediated rearomatization to furnish trifluoromethylated aromatic products (Scheme 10A; Campbell et al., 2024). This LMCT-driven platform exhibits broad functional group tolerance under mild conditions, enabling scalable synthesis of pharmaceuticals and advanced materials. Notably, the photoelectrochemical variant circumvents high catalyst loadings typical of conventional photoredox systems, showcasing a synergistic integration of light- and electricity-driven processes for energy-efficient bond activation.

Niu, Li, and Lan recently reported a visible-light-driven photocatalytic fluorotrifluoromethylation of unactivated olefins using Fe(acac)_3_ as the catalyst, TFAA as the CF_3_ source, and Selectfluor as the fluorine donor (Scheme 10B). Mechanistic studies revealed that the active species is an in situ-formed Fe(III) complex with CF_3_CO_2_^−^, H^+^, and acetonitrile, where Brønsted acids enhance LMCT efficiency via hydrogen-bonding interactions. Upon visible-light irradiation, LMCT excitation triggers decarboxylation of the Fe(III) complex, generating CF_3_• radicals and reducing Fe(III) to Fe(II). The CF_3_• radicals add to unactivated olefins, forming a carbon-centered radical intermediate, which abstracts a fluorine atom from Selectfluor to yield fluorotrifluoromethylated products. The catalytic cycle is sustained through two pathways: direct Fe(II) reoxidation by intermediate **II** (Path B) or via a diaryl ether-derived cation radical generated by intermediate **II**-mediated oxidation (Path A) (Jiang et al., 2024).

LMCT catalysis has emerged as a powerful strategy for trifluoromethylation under mild conditions, leveraging high-valent metal complexes (e.g., Fe(III), Ag(II)) to generate reactive CF_3_CO_2_• radicals via photoinduced O–M bond cleavage. These systems exhibit broad substrate scope and operational simplicity, bridging the gap between photoredox and transition-metal catalysis. While challenges in regioselectivity control and catalytic turnover efficiency persist, their ability to enable sustainable transformations underscores LMCT’s potential for mechanistic innovation. This progress highlights LMCT’s transformative role in radical chemistry, prompting further exploration of scalability and selectivity in next-generation synthetic methodologies.

## 3 Conclusion

This review provides an overview of the recent advancements in photocatalytic decarboxylative trifluoromethylation using TFA and its derivatives at room temperature, with a detailed exploration of three major catalytic systems: SET, EDA, and LMCT. While the SET process has enabled the oxidation and decarboxylation of TFA to some extent, the use of high-energy light sources (such as tungsten, mercury, and UV lamps) has led to lower reaction selectivity. Although noble metal catalysts can activate TFA under visible light, their high cost hinders industrial applications. The EDA process, which induces electron transfer through the formation of electron donor-acceptor complexes under light irradiation, has mitigated some of the stringent conditions required for intermolecular electron transfer but still needs optimization to enhance efficiency and selectivity. The LMCT process, particularly the photoinduced homolysis reactions based on 3d transition metals (e.g., iron), offers a sustainable alternative for visible-light-driven TFA activation, demonstrating the potential for activating high-redox-potential molecules under mild conditions. However, research on this system remains limited, and further expansion to a broader range of transition metal catalysts and applications is needed.

Despite significant progress in the field of TFA and its derivatives for decarboxylative trifluoromethylation, numerous challenges and opportunities remain. On one hand, the development of more efficient, green, and selective catalytic systems remains a key research focus. Future efforts could explore novel ligands or catalyst structures to optimize the LMCT process, enhance the generation efficiency of trifluoromethyl radicals, and thereby improve the activity and selectivity of the reactions. On the other hand, the photoinduced multi-photon redox process, although showing promise, is still in its developmental stage. A deeper understanding of its reaction mechanisms and exploration of how to more precisely control the photon absorption process are needed to achieve the transformation of high-redox-potential and high-bond-energy molecules under milder conditions. Additionally, expanding the substrate scope beyond olefins and arenes to other types of compounds for trifluoromethylation reactions is an important direction to further enrich the scope of organic synthesis. Simultaneously, integrating theoretical calculations with experimental studies will aid in a deeper understanding of the microscopic mechanisms of electron transfer and energy transfer in the reaction processes, providing theoretical guidance for designing more rational catalytic systems. As research continues to advance, it is anticipated that trifluoromethylation reactions will play an increasingly important role in the field of organic synthesis, offering more compounds with unique properties for applications in drug development, materials science, and beyond.
